# Pathway design using de novo steps through uncharted biochemical spaces

**DOI:** 10.1038/s41467-017-02362-x

**Published:** 2018-01-12

**Authors:** Akhil Kumar, Lin Wang, Chiam Yu Ng, Costas D. Maranas

**Affiliations:** 1 The Huck Institutes of the Life Sciences, The Pennsylvania State University, University Park, PA 16802 USA; 2 Department of Chemical Engineering, The Pennsylvania State University, University Park, PA 16802 USA

## Abstract

Existing retrosynthesis tools generally traverse production routes from a source to a sink metabolite using known enzymes or de novo steps. Generally, important considerations such as blending known transformations with putative steps, complexity of pathway topology, mass conservation, cofactor balance, thermodynamic feasibility, microbial chassis selection, and cost are largely dealt with in a posteriori fashion. The computational procedure we present here designs bioconversion routes while simultaneously considering any combination of the aforementioned design criteria. First, we track and codify as rules all reaction centers using a prime factorization-based encoding technique (rePrime). Reaction rules and known biotransformations are then simultaneously used by the pathway design algorithm (novoStoic) to trace both metabolites and molecular moieties through balanced bio-conversion strategies. We demonstrate the use of novoStoic in bypassing steps in existing pathways through putative transformations, assembling complex pathways blending both known and putative steps toward pharmaceuticals, and postulating ways to biodegrade xenobiotics.

## Abstract (web summary)

Existing pathway design tools make use of existing reactions from databases or successively apply retrosynthetic rules. novoStoic provides an integrated optimization-based framework combining known reactions with novel steps in pathway design allowing for constraints on thermodynamic feasibility, product yield, pathway length and number of novel steps.

## Introduction

Advances in genetic engineering capabilities have expanded the range of chemicals synthesized by microbial platforms to non-natural synthetic molecules such as drugs and various pharmaceutical precursors. Synthesis of these non-natural molecules often relies on enzymes with broad-substrate range^1, 2^ as well as enzymes with promiscuous activity on novel substrates^3^. A recent survey of *Escherichia coli* enzymes revealed that 37% of them could act on multiple substrates^4^. Protein engineering techniques have already demonstrated the feasibility of expanding the substrate range of existing enzymes^5–8^. These examples allude to the increasingly important role of in silico protein modeling tools such as IPRO^9^ and Rosetta^10^ to systematically change enzyme substrate specificity. In addition, recent successes in the de novo enzyme design^11, 12^ have expanded the scope of enzymatic functions that can be called upon in the construction of synthetic de novo metabolic pathways. Nevertheless, the systematic redesign of enzymes for new and novel activities remains a daunting challenge implying that novel transformations should only be sparingly used only if they confer pathway length, carbon yield, and/or redox benefits.

Computational pathway design and retrosynthetic tools can be used to guide the systematic recruitment of either native or de novo enzymatic functions to assemble pathways toward targeted chemicals. This is an area of research with significant prior work. Network-based path finding methods such as PathComp^13^, Pathway Hunter Tool^14^, and MetaRoute^15^ identify linear pathways from a single source to one target molecule. These methods rely on heuristics such as substrate-product similarity, atom transitions, or substrate-product reaction co-occurrence frequency to reduce carbon loss while designing the path from source to target. In contrast to linear pathfinding methods, network optimization-based approaches such as CFP^16^, k-shortest EFM^17^, and optStoic^18^ can incorporate non-injective (i.e., not necessarily a one-to-one mapping between reactants to products) stoichiometry and directly model carbon flow as well as cofactor balancing. All network-based path finding methods require literature extracted information available in biochemical databases such as MetRxn^19^, KEGG^20^, BRENDA^21^, and MetaCyc^22^. Prediction methods, on the other hand, expand upon uncovered knowledge space by suggesting putative reactant combinations plausible under the tenets of organic chemistry. By drawing from biochemistry principles, atom connectivity changes, or molecule fingerprint changes between substrates and products have been encoded as reaction rule operators in formats such as BEM^23^, RDM^24^, and SMIRKS^25^. Pathway prediction techniques such as BNICE^26^, XTMS^27^, UM-PPS^28^, PathPred^29^, Route Designer^30^, and GEM-Path^31^ employ reaction rule operators for a single molecular target iteratively in a retrosynthetic fashion so as to identify a bioconversion from a single source. This traversal strategy invoked by such retrosynthesis algorithms prunes the vast combinatorial space of putative transformations by evaluating free-energy change and substrate similarity metrics at each iteration. However, after each step, the trade-off between carbon yield, energy (ATP requirements), and thermodynamic feasibility of the main carbon conversion path often remain unexplored. Instead, tools such as TMFA^32^ and EFM^33^ are used in a posteriori steps to assess energy and carbon efficiency. Computational tools such as optStoic^18^ can be used to design mass and energy balanced pathways. However, they are limited to using reactions present in existing biochemical databases and metabolic models. Therefore, invoking novel molecules as intermediates through hypothetical reactions while maintaining a mass and energy balanced pathway remains elusive.

**Fig. 1:**
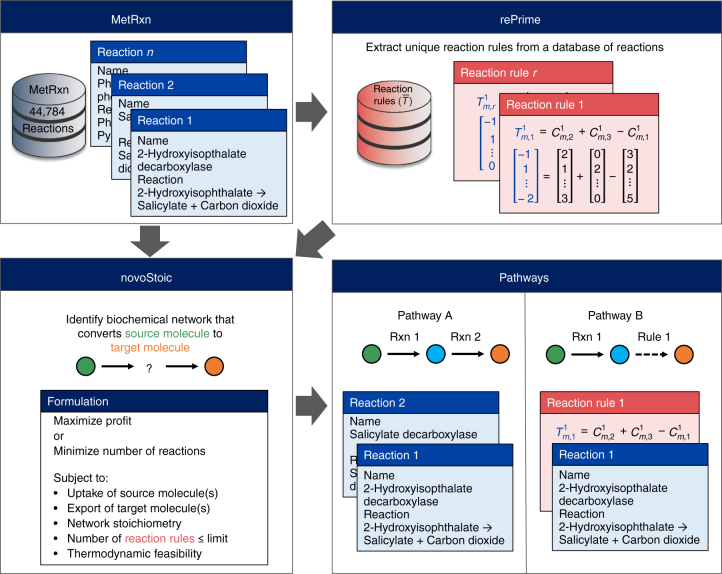
Schematic overview of the rePrime/novoStoic procedure. First, the rePrime procedure is used to pre-process the MetRxn database of reactions (blue boxes) to extract a unique set of reaction rules, *R*^*λ*^ (red boxes) at moiety size *λ*. The reaction rules are derived from the molecular signature $C_{mi}^\lambda$ of each participating metabolites and are captured by $T_{mr}^\lambda$. The novoStoic procedure is then used to identify a series of intervening reactions and reaction rules that convert source molecule(s) (green circle) into target molecule(s) (orange circle) such that the profit can be maximized or the number of reactions in the pathway can be minimized. Other criteria including the number of reaction rules and thermodynamic feasibility of the pathway can be flexibly incorporated as constraints. By controlling the number of reaction rules, two possible pathways designed by novoStoic are shown in the bottom right panel. Pathway A uses two known reactions (blue boxes) that present in the MetRxn database, whereas pathway B uses a combination of a known reaction (blue box) and a reaction rule (red box) to perform the same conversion

To address the problems in current computational methods, herein we present rePrime and novoStoic, an optimization-based de novo pathway design framework that seamlessly integrates existing reactions and novel reaction rules. First, we augmented the MetRxn repository^19^ with a new data set of elementally balanced reaction operators using the automated CLCA-^34^ based reaction rule extraction procedure termed rePrime. For each reaction, the rePrime procedure identifies and captures as a reaction rule the molecular graph topological changes underpinning the substrate to product graph conversion. A reaction rule is a vector that captures the location of active reaction centers affected by the conversion of substrates to products. The reaction rules and known reactions are then operated upon a mixed integer linear programming (MILP) procedure, novoStoic that identifies a mass-balanced biochemical network that converts a source metabolite to a target while satisfying a multitude of constraints and optimizing the objective function. Both precursors and possibly co-substrates and co-products required to satisfy the mass balance constraints serve as optimization variables (see Fig. 1 for a schematic overview). Overall, novoStoic identifies “by design” mass-balanced, high yield, and economically favorable biotransformations with a negative overall standard Gibbs free energy change from a substrate(s) to natural and synthetic product(s) by combining both existing and novel reactions. novoStoic can also be used to elucidate strategies to biodegrade xenobiotics by targeting a broad activity toward a topologically diverse set of substrates. In the Results sections, we introduce four case studies of different complexities, i.e., an illustrative example of rePrime/novoStoic procedure, the biosynthesis of 1,4-butanediol (bdo), the synthesis of phenylephrine, and the biodegradation of benzo[a]pyrene.

## Results

### An illustrative example for rePrime and novoStoic

**Fig. 2:**
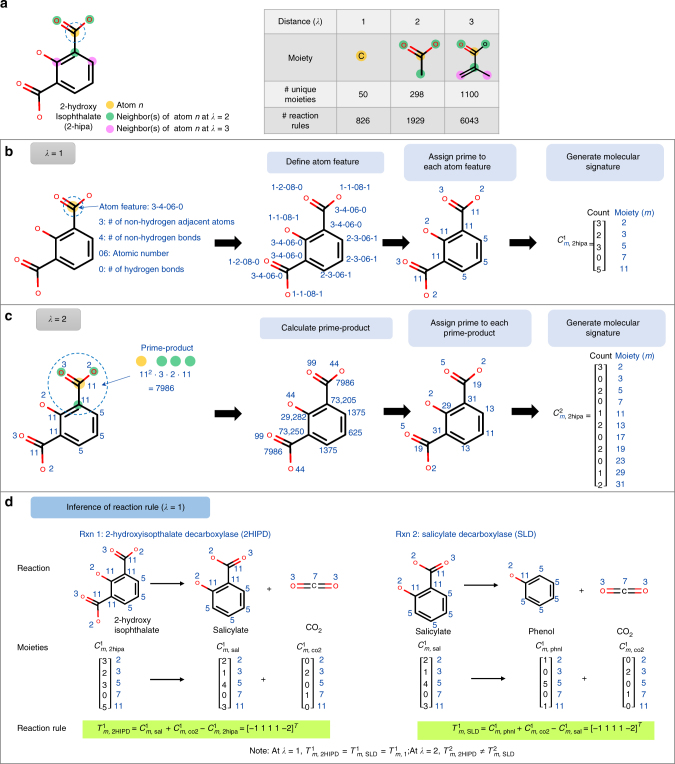
The rePrime procedure is demonstrated for the molecule 2-hydroxy isophthalate (2hipa). **a** The moiety size (*λ*) indicates the distance between nodes in a molecular graph. At *λ* = 1, the moiety at node *n* (the carbon atom shaded in orange) is the atom itself. The moiety centered at node *n*, the number of unique moieties and reaction rules extracted from MetRxn at $\lambda = \{ 1,2,3\}$ are provided on the table. **b**,** c** The rePrime procedure iteratively assigns prime number to each node at *λ* = 1, 2 (see Supplementary Methods for details). The molecular signature of 2hipa, $C_{m,\rm{2hipa}}^\lambda$ contains the count of unique moieties (i.e., prime number). The resulting $C_{m,\rm{2hipa}}^\lambda$ increases in specificity at larger moiety size (*λ*) as neighboring atoms are also involved in determining the canonical label of the node. **d** The derivation of reaction rule for two decarboxylase reactions (i) 2-hydroxyisophtalate decarboxylase (2HIPD) and (ii) salicylate decarboxylase (SLD) are demonstrated at *λ* = 1. The resulting reaction rules $T_{m,\rm{2HIPD}}^1$ and $T_{m,\rm{SLD}}^1$ are identical at this moiety size, hence they are stored as $T_{m,1}^1$. The specificity of the reaction rules increases with increasing moiety size *λ*, therefore $T_{m,\rm{2HIPD}}^2$ is not equal to $T_{m,\rm{SLD}}^2$ (Supplementary Table 3)

We first clarify the rePrime and novoStoic procedures using only two decarboxylase reactions (i) 2-hydroxyisophthalate decarboxylase (2HIPD) and (ii) salicylate decarboxylase (SLD), and four participating metabolites. The first step (rePrime) extracts reaction rules from a database of known reactions (Fig. 1). To this end, each metabolite *i* within the database is first encoded using a molecular signature (see Supplementary Methods for detail procedure). For a moiety size *λ*, a molecular signature $\left( {C_{mi}^\lambda } \right)$ is a vector that concatenates into a single numeric value the number of attributes for every moiety *m* in a metabolite *i*, described as a collection of prime numbers centered at node *n*. For *λ* = 1, the moiety is simply the atom at node *n*, whereas at *λ* = 2 the moiety is composed of all the atoms bonded to the atom at node *n* (Fig. 2a). Figure 2b, c shows the iterative rePrime procedure to generate molecular signature at moiety size *λ* = 1 and 2. In brief, each iteration involves the assignment of a canonical label followed by a prime number assignment to each node. The unique prime numbers are then counted and stored in the molecular signature vector. We apply the rePrime algorithm to generate the molecular signatures for metabolites 2-hydroxyisopthalate (2hipa), salicylate (sal), carbon dioxide (CO_2_), and phenol (phnl) at different moiety sizes *λ* (see Fig. 2b, c and Supplementary Tables 1, 2, and 3). A reaction rule $\left( {T_{mj}^\lambda } \right)$ is defined as a vector that captures the changes in all moieties *m* of the participating metabolites upon reaction *j*. It is derived based on the reaction stoichiometry and the corresponding molecular signatures of the reactants and products. A reaction rule uniquely captures the eliminated and newly formed moieties around the reaction center. In Fig. 2d, we generated the reaction rules for reactions 2HIPD and SLD for moiety size *λ* = 1. Upon removing repetitive rules, a unique set of reaction rules are indexed by set *R*^*λ*^ (i.e., $T_{mr}^\lambda$). Hence, $T_{m,2{\mathrm{HIPD}}}^1$ and $T_{m,{\mathrm{SLD}}}^1$ are now stored as $T_{m,1}^1$.

**Fig. 3:**
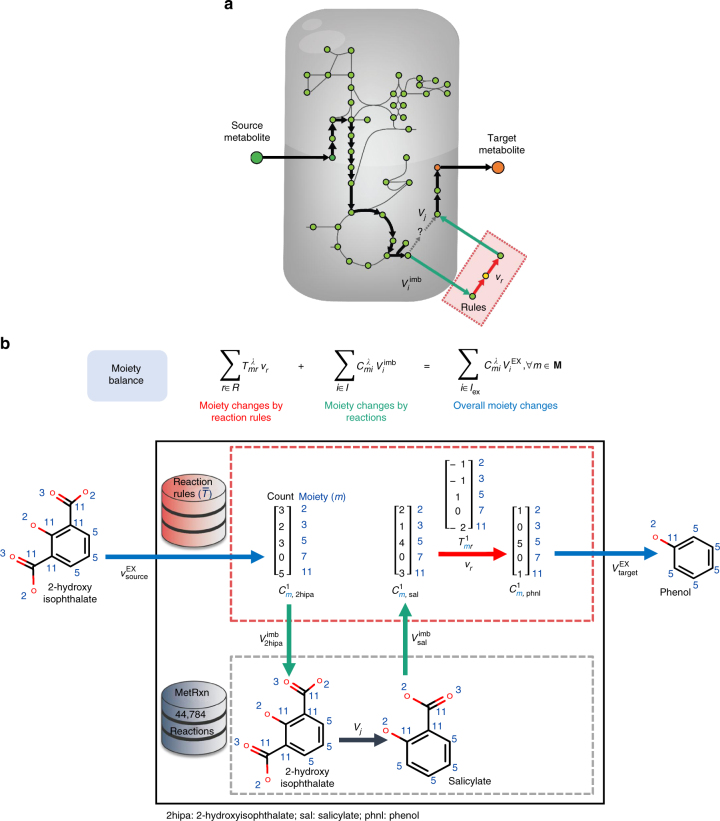
The novoStoic procedure. novoStoic seamlessly blend existing reactions and reaction rules while designing a pathway from source metabolite to target metabolite. **a** The variable $v_i^{\rm{imb}}$ defines the connection (green arrows) between the known reactions network (black arrows) and the reaction rules network (red arrows and red dotted box). It indicates the surplus or deficit of metabolite *i* in the known metabolic network. The reaction rules network is invoked to complete a pathway when no known reaction can catalyze the conversion. **b** Illustration of the component balance and moiety balance defined in novoStoic constraints (2) and (3) (Supplementary Methods). The bottom half (gray dotted box) is the known network operating on metabolites, while the upper half (red dotted box) is the reaction rules network operating on moieties. The section of the entire system that is involved in the moiety balance constraint is color-coded: (red) moiety changes by reaction rules, (blue) overall moiety changes, and (green) moiety changes by known reactions. The pathway here converts 2hipa to phnl using a known reaction (i.e., 2hipa → sal, black arrow) and a reaction rule (i.e., sal → phnl, red arrow). Metabolite 2hipa first enters the reaction rule network using exchange reaction (blue arrow, $v_{\rm{source}}^{\rm{EX}}$) and subsequently exported to the known reaction network (green arrow, $v_{\rm{2hipa}}^{\rm{imb}} = - 1$). It is converted by a known reaction (black arrow) into sal, which is then transferred to the reaction rule network (green arrow, $v_{\rm{sal}}^{\rm{imb}} = 1$). The reaction rule $T_{mr}^1$ (red arrow) converts sal into phnl, which is exported out of the entire system $\left( {v_{\rm{target}}^{\rm{EX}}} \right)$. Note that for clarity purpose, the involvement of carbon dioxide in the conversion of 2hipa to sal and sal to phnl is omitted

The second step (novoStoic) uses an MILP representation to pose the task of identifying a component and moiety balanced biochemical pathway that converts a source to a target metabolite as an optimization problem. It searches the database of both known reactions and reaction rules to complete the pathway (Fig. 3) by introducing moiety balances along with component balances as key constraints in the MILP formulation. The two balances are linked through the imbalance metabolic flux *v*^imb^, which quantifies the surplus or deficit for any metabolites in the metabolic network that necessitates the involvement of novel reaction steps (Fig. 3a) to balance the overall conversion. The overall change for moiety *m* $\left( { {\sum }_{i \in I_{\rm{ex}}} C_{mi}^\lambda v_i^{\rm{EX}}} \right)$ is set to be equal to the moiety changes incurred in both the novel reaction network $\left( { {\sum }_{r \in R^\lambda } T_{mr}^\lambda v_r} \right)$ and the ones implied by the known metabolic reaction network $\left( { {\sum }_{i \in I} C_{mi}^\lambda v_i^{\rm{imb}}} \right)$ (Fig. 3b). Herein, we used novoStoic to design biosynthesis pathways from 2hipa to phnl and obtained four separate solutions. novoStoic not only identified a pathway which involves two known reactions 2HIPD and SLD but also pathways which involve a single reaction rule $\left( {T_{m,1}^1} \right)$ implying a novel conversion (see Supplementary Fig. 1 and Supplementary Fig. 2 for the details).

The toy example helps explain how the two key quantities molecular signatures $\left( {C_{mi}^\lambda } \right)$ and reaction rules $\left( {T_{mr}^\lambda } \right)$ are used to impose constraints for mass and moiety balance. In the following case studies, we will explore much more complex conversion strategies that make use of multiple novel reactions absent from the reaction databases. Each reaction rule identified is then matched to a corresponding enzyme homolog. Supplementary Methods provide a thorough description of the algorithmic details of rePrime and novoStoic along with a pseudo-code description.

### 1,4-butanediol synthesis

1,4-butanediol (bdo) is a commodity chemical that is used as an industrial solvent and as a monomer in polymer synthesis. Efforts are under way aimed at replacing petroleum-derived production with more sustainable processes using bio-based production based on genetically engineered microbial strains^35^. By leveraging computational design and scoring of a large number of bdo biosynthetic routes, Yim et al. reconstituted a pathway in *E. coli* with a titer of 18 g/L[35]. Herein, we demonstrate three pathways designed using KEGG database and our novoStoic algorithm, which seamlessly combines known and novel steps starting from the precursor succinyl-CoA (succoa) toward bdo. novoStoic provides possibilities for lowering the number of steps in the pathway by invoking novel steps.

**Fig. 4:**
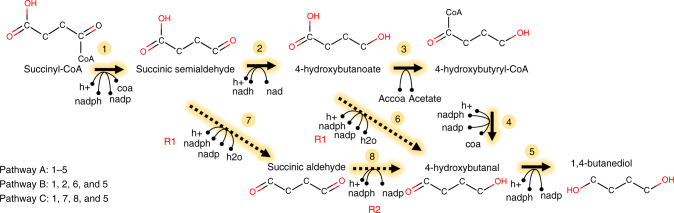
1,4-butanediol biosynthesis. The figure shows three routes from succinyl-CoA to 1,4-butanediol. Known reactions are indicated by solid lines while reaction rules are denoted with dashed lines. Every hypothetical reaction step is indexed with a reaction rule id shown in red (see Table 1 for description)

Pathway 4A identified by novoStoic is a five-step pathway of bdo synthesis that recapitulates the downstream pathway of Yim et al.^35^ (Fig. 4, steps 1–5). This pathway converts succoa into bdo with a concomitant oxidation of four NAD(P)H and production of two co-enzyme A (coa) molecules. Co-substrate acetyl-coA is converted into acetate in the third step. The overall conversion of this pathway is given by

$$\rm{succoa} + {3\, \rm{NADPH}} + \rm{NADH} + 4\,H^ + + accoa\mathop{\longrightarrow}\limits^{{ - 49\,kcal/mol}}bdo \\ \rm + ac + 3\,NADP + NAD + 2\,coa.$$

Pathway 4A can be further improved upon by preventing carbon flux from draining toward undesirable co-substrates/co-products (i.e., accoa and acetate) when glucose is used as feedstock thereby leading to a higher theoretical carbon yield. Furthermore, we explored if a shorter pathway is feasible upon invoking one or two novel transformations. Therefore, we imposed three additional design criteria: (1) the fewest number of reaction rules, (2) the maximal number of steps from succoa to bdo is less or equal to four, and (3) no acetyl-CoA as a co-substrate. Criterion (1) was set as the objective function and criteria (2) and (3) were defined as constraints in the novoStoic MILP formulation. As a result, novoStoic identified pathway 4B that requires only four reaction steps (Fig. 4). Pathway 4B shares three reactions with pathway 4A (steps 1, 2, and 5). In a departure from pathway 4A, intermediate 4-hydroxybutanoate generated by step 2 is directly converted into 4-hydroxybutanal, thus bypassing steps 3 and 4, using reaction rule R1 (Fig. 4; Table 1). A putative succinate-semialdehyde dehydrogenase is suggested as the enzyme homolog for this step. The overall conversion can be described as followed:

$$\mathrm{succoa} + 3\,\mathrm{NADPH} + \rm {NADH} + 4\,H^ + \mathop{\longrightarrow}\limits^{{ - 52\,kcal/mol}}bdo \\ \rm+ 3\,NADP + NAD + coa + H_2O.$$

**Table 1:** Reaction templates for 1,4-butandiol (bdo) synthesis

The reaction-rule template column presents the reactions that correspond to the reaction rules identified by the alphanumeric scheme (i.e., R1 and R2) for 14bdo synthesis. Step id identifies the reaction that was predicted from the corresponding reaction rule

Pathway 4C was identified by novoStoic upon appending the constraint of using a single cofactor (i.e., NADPH). Reducing the number of cofactors can help focus the strain engineering efforts on only increasing NADPH availability without having to worry about the balance of the stoichiometric ratio NADH:NADPH while improving both their availabilities.

$$\mathrm{succoa} + 4\,\mathrm{NADPH} + 4\,\mathrm{H}^ + \mathop{\longrightarrow}\limits^{{ - 52 {\, kcal/mol}}}\mathrm{bdo} + 4\,\mathrm{NADP} \\ + {\rm coa} + {\rm H_2O}.$$

This exact pathway was identified before by GEM-path^31^ and found to be exhibiting higher theoretical product yield using FBA. In contrast to pathway 4B, two NADPH-dependent novel steps are required in this pathway, in order to bypass the NADH-dependent 4-hydroxybutyrate dehydrogenase (step 2). The direct production of succinic aldehyde from succinic semialdehyde (product of step 1) was proposed by novoStoic by invoking a homolog of succinate-semialdehyde dehydrogenase (R1). The homolog of 4-hydroxybutryate dehydrogenase (R2) was next suggested for the conversion of succinic aldehyde to 4-hydroxybutanal. In all three pathways, the intermediate 4-hydroxybutanal is produced in the second to last step, which is then converted into bdo using alcohol dehydrogenase (step 5).

Despite requiring the same numbers of redox cofactors, pathways 4B and 4C designed by novoStoic bypass the involvement of acetyl-CoA and generation of the by-product acetate. We caution the reader that computational pathway design using novoStoic requires careful scrutiny of the cofactor systems as there are often inconsistencies between current databases and experimental studies. For example, reaction rule R1 was reported to utilize ATP as a cofactor^36^, whereas the manually approved Rhea database^37^ and KEGG^20^ denote it as a reversible reaction without utilization of ATP. When we enforced rule R1 to use ATP (Supplementary Tables 5, 6), novoStoic designed a pathway with a different overall stoichiometry including ATP as one of the cofactors:

$$\mathrm{ATP} + \mathrm {succoa} + 4\,\mathrm{NADPH} + 4\, \rm H^ + \mathop{\longrightarrow}\limits^{{{\mathrm - 58\,kcal/mol}}}bdo \\ \rm+ 4\,NADP + coa + ADP + pi.$$

This small molecule focused example demonstrates the potential of novoStoic to handle multiple constraints on pathway design and bypass multiple known steps by invoking novel conversions only when needed.

### Phenylephrine synthesis

**Fig. 5:**
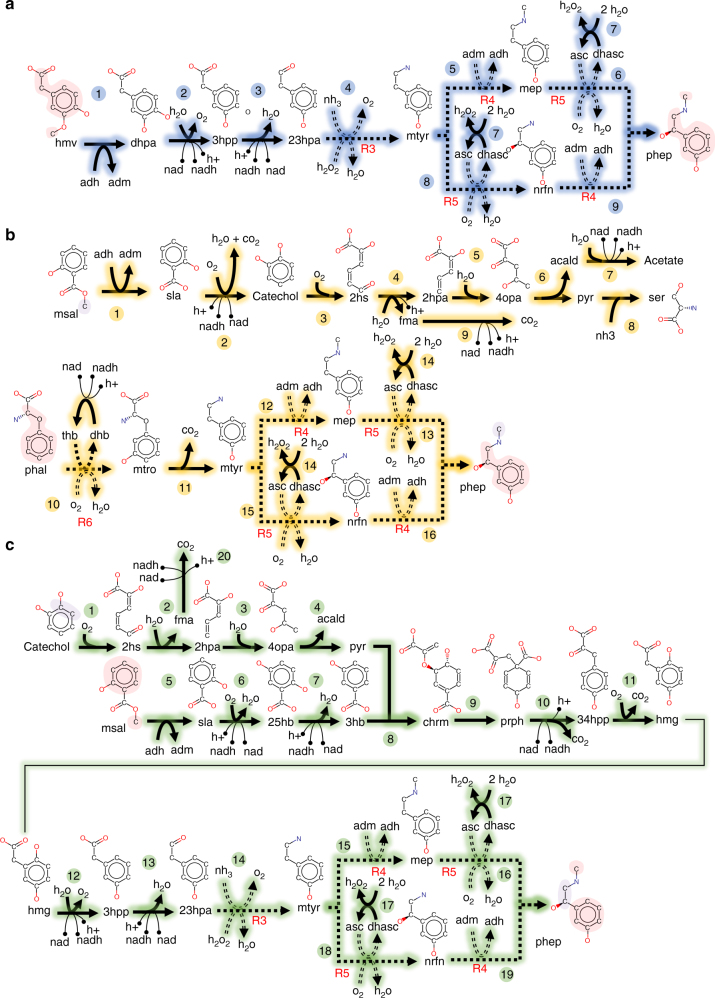
Phenylephrine synthesis. **a**–**c** Three routes are depicted for the synthesis of the phenylephrine from benzoate derivatives. Each route is color coded to differentiate the route by choice of precursor and overall conversion. In addition, the precursor and target molecules are colored to depict moiety translocation. Known reactions are indicated by solid lines while reactions suggested using reaction rules are denoted with dashed lines. Every hypothetical reaction step is indexed with a reaction rule id (see Table 2 for description). The structure for pyruvate (pyr), acetaldehyde (acald), acetate, carbon dioxide (CO_2_), oxygen (O_2_), hydrogen peroxide (H_2_O_2_), formate (fma), *S*-adenosyl-l-methionine (adm), *S*-adenosyl homocysteine (adh), ascorbic acid (asc), dehydroascorbic acid (dhasc), tetrahydrobiopterin (tdh), and 4a-hydroxytetrahydrobiopterin (dhb) are not shown for clarity purpose

The non-natural molecule phenylephrine (phep) is a member of the phenylethanolamines class. It mimics the action of stimulants such as adrenaline and dopamine. Traditionally, phenylephrine is produced through chemical synthesis involving the reduction of the aromatic substrate m-hydroxybenzaldehyde^38^. Figure 5 illustrates three pathways each starting with a different aromatic precursor with B and C identified by novoStoic. The pathways involve (i) homovanillate (hmv) as the only substrate, (ii) co-utilization of phenylalanine (phal) and methyl salicylate (msal), and (iii) catechol along with methyl salicylate.

Pathway 5A is an example of a design with a positive standard Gibbs free energy of change (Fig. 5a). It involves three existing reactions (steps 1–3) and three reaction rules (R3, R4, and R5 in steps 4–9) to perform the overall conversion as followed:

$$\mathrm{hmv} + \rm NH_3\mathop{\longrightarrow}\limits^{{ + 127\,kcal}}phep + O_2.$$

It is a solution typically predicted by substrate similarity-based retrosynthesis tools. Such algorithms first choose a likely substrate as the target feedstock and then proceed to prune the retrosynthesis reaction network using similarity-based filters on each backward reaction step until the shortest (one substrate to one product) linear route is found. The free energy change in the direction of bioconversion toward phep is positive and thus thermodynamically infeasible.

Pathway 5B involves the conversion of co-substrates phal and msal to phep with a concomitant production of l-serine (ser) and acetate (Fig. 5b). The overall conversion for pathway 5B is:

$$\mathrm{phal} + \rm msal + 2\,H_2O + 4\,O_2 + NH_3\mathop{\longrightarrow}\limits^{{ - 323\,kcal}}ser + phep \\ \rm+ acetate + 3\,CO_2 + H_2O_2.$$

In the first half of the 14 reaction cascade (steps 1–9), the salicylate produced upon the demethylation of msal is converted to acetate and pyruvate via the benzoate degradation pathway. Pyruvate is then aminated by serine hydratase to produce l-serine. The degradation of msal to l-serine and acetaldehyde also produces an *S*-adenosyl-l-methionine (adm), which acts as a methyl donor in the second half of the network (steps 10–16). In addition, the NADH consumed in steps 2 and 10 is regenerated in steps 7 and 9, thereby maintaining the NADH/NAD^+^ cofactor balance.

In the second half of the network, the phal to phep conversion starts with the reduction of phenylalanine by a homolog of phenylalaninase (step 10). A typical phenylalaninase hydroxylates phal at the 4th carbon position of the phenyl ring to produce *p*-tyrosine (Table 2, R6). However, to produce *m*-tyrosine (mtro), a hydroxylation at the 3rd carbon position is suggested (step 10). This reaction is not present in any of the reaction databases and is predicted de novo by novoStoic. Nevertheless, pacidamycin studies focusing on bacterial phenylalaninase have indicated the presence of the phenylalaninase homolog in many *Streptomyces* species with regiospecific hydroxylation activity at the 3rd carbon atom of the phenyl ring^39^. For example, the phal to *m*-tyrosine reaction suggested by novoStoic is predicted as a secondary activity by the phenylalanine hydroxylase homolog encoded by *pacX* from *Streptomyces coeruleorubidus*^39^.

**Table 2:** Reaction templates for phenylephrine synthesis

*S*-adenosyl-L-methionine (adm), *S*-adenosyl homocysteine (adh), ascorbic acid (asc), dehydroascorbic acid (dhasc), tetrahydrobiopterin (tdh) and 4a-hydroxytetrahydrobiopterin (dhb)

Step 11 involves the decarboxylation of *m-*tyrosine to form *m*-tyramine catalyzed by *m*-tyrosine decarboxylase (EC 4.1.1.28) (Fig. 5b). The conversion of *m*-tyramine to phep (steps 12, 13, 15, and 16) in the remainder of the network is identical to the subnetwork of pathway 5A with the same *N*-methyltransferase (R4) and oxidoreductase (R5) reactions. With msal contributing only one carbon as a methyl group toward phep synthesis, we can, therefore, consider only the second half (steps 10–14) of the network for phep if endogenous adm is supplied.

Pathway 5C shown in green in Fig. 5c combines a number of fungal reactions from the tyrosine and phenylalanine metabolism pathways for the conversion of catechol and methyl salicylate to acetaldehyde and phenylephrine. This pathway is similar to the phytochemical synthesis pathway of pseudoephedrine, wherein the carboligation product of the benzoate derivate and pyruvate undergoes transamination and subsequently *N*-methylation^40^. The overall conversion for pathway 5C is:

$$\mathrm{catechol} + \rm msal + 2\,O_2 + H_2O + NH_3\mathop{\longrightarrow}\limits^{{ - 60\,kcal}}phep \\ \rm+ acetaldehyde + 2\,CO_2.$$

Two possible routes with 17 reactions each were suggested (Fig. 5c). novoStoic predicted a novel reaction (R3) to convert 23hpa to *m*-tyramine as the next step (step 14), which requires a homolog of monoamine oxidase. The monoamine oxidase typically deaminates *p*-tyramine, with trace deamination activity reported for *m-*tyramine^41, 42^. Similar with pathway 5A and 5B, the conversion of *m*-tyramine to phenylephrine in the last two steps proceeds further by the action of an *N*-methyltransferase (R4) and oxidoreductase (R5). The de novo steps suggested by novoStoic provide the reaction templates for the development of protein engineered dopamine hydroxylase (R5, steps 16, 18) and phenylethanolamine methyltransferase (R4, steps 15, 19) that can act upon new substrates.

The co-substrate *S*-adenosyl-l-methionine (adm) required by the *N*-methylation reaction is generated in the *O*-methylation reaction by the action of salicylate 1-*O*-methyltransferase (step 5). Therefore, msal provides the carbons in the phenolic and the methylamino moieties, whereas catechol provides the carbons in the ethyl moiety of phenylephrine through its degradation to pyruvate (Fig. 5c). Steps 1–4 can be bypassed if pyruvate is directly provided. Note that the reaction in step 13 is treated as reversible in accordance with the reaction designation in the source database MetRxn without requiring ATP. However, literature evidence^36^ suggests that step 13 requires ATP. The updated overall stoichiometry with ATP as a cofactor for step 13 becomes:

$$\rm ATP + catechol + msal + 2\,O_2 + 2\,H_2O + NH_3\mathop{\longrightarrow}\limits^{{ - 66\,kcal}}phep \\ \rm+ acetaldehyde + 2\,CO_2 + ADP + pi.$$

The total number of steps of the designed pathways is generally larger than the synthetic chemistry-based approaches^43–45^. However, if the methyl donor adm can be provided by the host cell (e.g., engineered *Saccharomyces cerevisiae*^46^ and *Pichia pastoris*^47^), then pathway 5B can be shortened into four steps thereby becoming on par with the synthetic chemistry pathways. It is noteworthy that the identified pathways use a more appealing set of substrates (e.g., catechol and methyl salicylate) with a lower cost than the substrate (i.e., *m*-hydroxybenzaldehyde) commonly used for chemical synthesis. In all the three pathways designed, the conversion of the intermediate *m*-tyramine to phenylephrine involves reaction mechanisms that can be derived from natural enzymes thus suggesting potential candidates for protein engineering (Table 2). In the next case study, we focus on identifying biodegradation pathways using reaction rules from a particular organism or species (see Supplementary Methods, novoStoic design criteria iii/iv).

### Oxidative degradation of benzo[a]pyrene to catechol

**Fig. 6:**
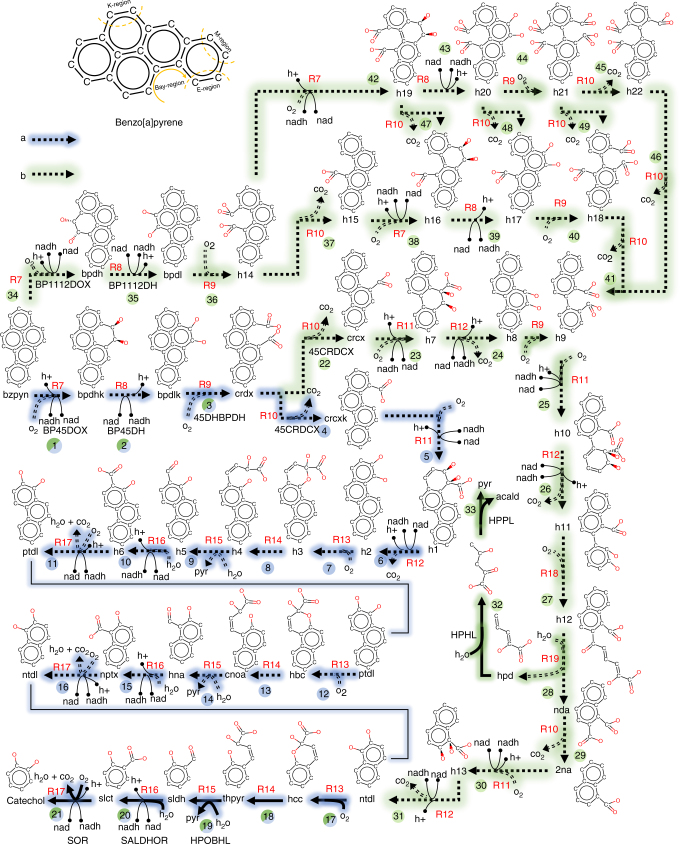
Benzo[a]pyrene degradation pathway. The oxidative degradation routes suggested by novoStoic combine both existing reactions and reaction rules in a mass-balanced fashion. The degradation products pyruvate and catechol were set as targets and benzo[a]pyrene was set as the source metabolite. Known reactions are indicated by solid lines while reactions suggested using reaction rules are denoted with dashed lines. Reaction rule ids are shown in red (see Table 3 for description). Novel intermediates predicted by novoStoic are abbreviated using an alphanumeric scheme (i.e., h1, h2, h3, etc.). The predicted routes are color-coded in blue and green based on the overall conversion. The degradation initiates with the formation of a (poly)aromatic diol in a dioxygenase reaction (R11–R12), while a subsequent oxidation forms a heterocyclic chromene derivative (h3). Next, a ring opening reaction of the chromene derivative (R14) is followed by an aldolase reaction to yield pyruvate and a (poly)aromatic aldehyde (R15). The aldehyde is then oxidized yielding carbon dioxide and a (poly)aromatic diol as a substrate for the next decyclization process (R16, R17)

**Fig. 7:**
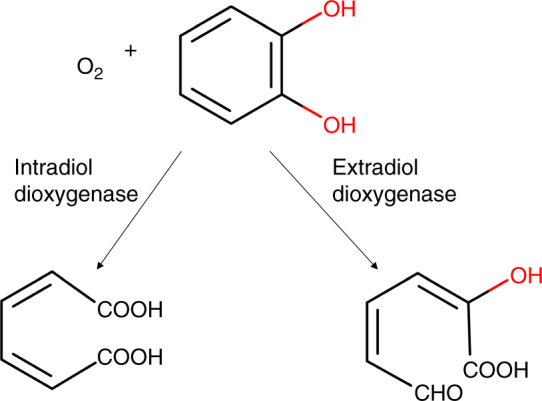
Intradiol and extradiol cleavage. Intradiol dioxygenases catalyze the decyclization reactions at *ortho-*positions, while extradiol dioxygenases catalyze the decyclization reactions at *meta-*positions

Polycyclic aromatic hydrocarbons (PAHs), with mutagenic and carcinogenic properties, are unfortunately ubiquitous in the environment and have both natural and anthropogenic origins^48^. Biodegradation studies around industrial effluent treatment plants and hydrocarbon drilling sites have implicated PAHs as the sole carbon and energy source for many soil dwelling organisms^49^. Metabolic assays on a number of terrestrial bacterial species indicate the involvement of a common cluster of metabolic genes to convert various PAHs to metabolites of the central carbon pathways^50^. Numerous studies have identified various pathway intermediates and pointed toward the use of ring-cleaving dioxygenases to degrade various PAHs into pyruvate, catechol, and phthalate^51^. The dioxygenases first introduce oxygen to the aromatic rings (i.e., dioxygen activation), which are subsequently hydroxylated^52^. Decyclization reactions at the *ortho-*positions involve intradiol dioxygenases to cleave the carbon–carbon bonds between the two hydroxyl groups (e.g., R9 in Fig. 6), while the decyclization reactions at *meta-*positions involve extradiol dioxygenases to cleave carbon–carbon bonds adjacent to one of the hydroxyl groups (Fig. 7). When the substrate undergoes *ortho-*cleavage upon dioxygen activation, dehydrogenase, dioxygenase, and decarboxylase reactions enable the release of K-region carbons (Fig. 6) as two carbon dioxide molecules. However, when the substrate undergoes *meta*-cleavage, a pyruvate and carbon dioxide molecule is released for each decyclization step.

Using novoStoic, we considered both cleavage strategies while suggesting multiple catabolic routes for benzo[a]pyrene (bzp) to catechol. In addition, we also factored into our pathway design the findings from multiple metabolic PAHs degradation studies on various species. Metabolic studies on the *Pseudomonas* indicate the recruitment of *nah* genes and its homologs associated with naphthalene degradation in converting diverse bay-region PAHs to pyruvate through the naphthalene degradation pathway intermediates 1-naphthol-2-carboxylate and salicylaldehyde^53^. Thus, we focused our design rules toward reaction rules related to naphthalene degradation pathways by setting $y_{\mathit{nah}}^{\mathrm{path}} = 1$, $v_{\mathrm{catechol}}^{\mathrm{EX}} \ge 1,$ and $v_{\mathrm{pyr}}^{\mathrm{EX}} \ge 1$. In this way, we ensured that at least one molecule of catechol and pyruvate should be produced from each molecule of benzo[a]pyrene. We also imposed constraints (8) and (9) by setting $y_{Pseudomonas\,\mathrm{genus}}^{\mathrm{org}} = 1$ to study PAH biodegradation by only the organisms belonging to the *Pseudomonas* genus^53^. The reactions to genus associations were downloaded from KEGG. The benzo[a]pyrene degradation network to catechol is derived by limiting the list of co-substrates/products to central carbon metabolites and small molecules such as CO_2_, H_2_O, and O_2_.

By expanding upon the boundaries of catabolic routes to include cataloged intermediates and reactions, we identify the set of previously unknown reactions and provide a putative explanation for the complete degradation of benzo[a]pyrene to catechol. Figure 6 illustrates seven different routes from benzo[a]pyrene to catechol identified by novoStoic with the minimal number of hypothetical reaction steps (16 steps). The pathway design in blue contains one bioconversion route from benzo[a]pyrene to catechol while the grouping in green contains six different bioconversion routes. Our result recapitulates the findings of PAHs degradation through *ortho*-cleavage or *meta*-cleavage pathways and imputes metabolites into the ill-defined and incomplete benzo[a]pyrene degradation substrate annotations in current biochemistry databases.

Pathway 6A (Fig. 6, blue route) combines 21 reactions for the biodegradation of benzo[a]pyrene to catechol, pyruvate, and carbon dioxide. The overall stoichiometric conversion for Pathway 6A is:

$$\rm bzp + 8\,O_2 + 3\,H_2O \to catechol + 3\,pyr + 5\,CO_2.$$

The first 16 steps in the pathway are hypothetical reactions predicted by novoStoic. Reactions in steps 1–4 have incomplete EC numbers in KEGG. They were reproduced using reaction rules extracted from the reactions naphthalene dioxygenase (Table 3, R7), naphthalene dihydrodiol dehydrogenase (R8), protocatechuate oxidoreductase (R9), and phthalate decarboxylase (R10). Steps 5 and 6 were derived using reaction rules extracted from terephthalate dioxygenase (R11) and cyclohexadiene oxidoreductase (R12) reactions. Steps 1 and 2 are the dioxygen activation steps for *ortho*-cleavage, while steps 5 and 6 are dioxygen activation steps for *meta*-cleavage. Steps 7–11, 12–16, and 17–21 depict a cyclic pathway wherein a chromene derivative is produced (steps 7, 12, and 17) followed by the ring opening reaction (steps 8, 13, and 18). Next, a pyruvate molecule and an aromatic aldehyde are produced in steps 9, 14, and 19. The aromatic carboxylate generated in steps 10, 15, and 20 are subsequently decarboxylated to produce substrates (diols) for the next iteration of biodegradation. Steps 17–21 are known reactions indexed in current biochemistry databases. Additionally, of the 22 intermediate metabolites identified in this pathway, 16 are listed in most biochemistry databases.

**Table 3:** Reaction templates for benzo[a]pyrene

Note that reaction rules R13 to R17 are listed as step 17 to 21 in Fig. 6

Pathway 6B spans six separate routes, and they have the same stoichiometric overall conversion:

$$\rm bzp + 9\,O_2 + 3\,H_2O \to catechol + 2\,pyr + acld + 6\,CO_2.$$

While one of the routes in pathway 6B shares steps 1–3 with pathway 6A, all the routes share steps 17–21 with pathway 6A. Each route in pathway 6B requires the same number of 22 steps. In each route, the substrate undergoes two *ortho-*cleavage reactions and loses the K-region carbons as two carbon dioxide molecules, to converge at the hypothetical metabolite h9. Subsequently, h9 undergoes *meta-*cleavage to yield a pyruvate and an acetaldehyde molecule, as well as the naphthalene (ntl) degradation pathway intermediate, naphthalene 1,2-diol (ntdl). Ntdl is further degraded to catechol using steps 17–21.

novoStoic enables us to predict various intermediates and bioconversions and rapidly develop a hypothesis consistent with sparse information in many PAHs degradation studies and biochemical databases. The pathways predicted by novoStoic provide a complete overall stoichiometry of reactions from benzo[a]pyrene to catechol, while none of the current metabolic databases and metabolic models contain a complete degradation pathway. Based on the predicted degradation pathways we proposed here and a number of PAHs degradation studies, molecules such as chrysene and picene would not require any *ortho-*cleavage reactions to form naphthalene intermediates, while molecules such as pyrene and triphenylene would require at least one *ortho-*cleavage to form ntl intermediates.

## Discussion

In this paper, we introduce two novel procedures rePrime and novoStoic for the de novo pathway design. rePrime is a reaction rule-based algorithm that encodes reaction centers as elementally balanced operators. These reaction rules capture moiety changes in the reaction centers by using the changes in the counts of prime numbers (i.e., canonical label for moieties) between substrates and products. Other than metabolites currently present in the database, our approach can be extended to novel metabolites as long as the structure can be codified as counts of moieties (i.e., molecular signature). rePrime allows for different moiety sizes. In the current implementation of rePrime, we trace moieties of up to a size of *λ* = 3. In principle, one could expand the size of moieties traced or customize the size of the moiety traced based on the underlying reaction chemistry. By combining metabolite balance and moiety balance constraints, novoStoic simultaneously integrates reaction rules with known reactions. It thus enables homing in first to the most desirable designs avoiding costly enumeration of alternatives that either include too many novel steps, are redox imbalanced, or fail to meet cost/yield requirements. The MILP-based computational framework allows for straightforward control of cofactor regeneration, the number of novel reactions, and the imposition of carbon yield or profit margin requirements.

novoStoic allows us to exploit enzyme plasticity by suggesting homologs to perform the hypothesized conversion when natural options are not available. In typical industrial bioprocesses, the number of novel reactions must be carefully controlled (or minimized) as each novel reaction implies an additional enzyme–substrate engineering challenge. In the event that the homolog is not promiscuous, protein engineering steps have to be recruited to enhance non-natural substrate binding (e.g., by tuning the binding pocket structure to accommodate the non-natural substrate^54^) and subsequently to increase catalytic rate^55^. For example, Cargill, Inc. engineered a multi-step 3-hydroxypropionic acid biosynthesis pathway, which employed a single non-natural enzyme (i.e., alanine 2,3-aminomutase), to bypass an ATP consuming step^56^. The team had to engineer a homolog lysine 2,3-aminomutase to confer it the desirable activity and at the same time select a variant with the least negative effect on the host cell^57^. With the capability to blend known reactions and non-natural ones, novoStoic could invoke novel steps only when necessary.

A number of chemical manufacturing processes are increasingly exploiting the chemoselectivity and catalytic rate boost potential of enzymes^8, 58^ for the synthesis of pharmaceuticals and precursors. Studies have demonstrated that multi-enzyme cascades of non-natural enzymes can be implemented in both in vivo and in vitro fashion as well as in combination^59^. rePrime/novoStoic address the timely challenge of integrating recent advancements for the rapid identification of complete pathways for bio-based chemosynthesis and the elucidation of intermediates of ill-defined xenobiotic degradative pathways. The detailed degradation map can therefore assist in evaluating the toxicity and potential side effects of new drugs, and even enable the assessment of synergistic, antagonist, or toxic drug interactions.

novoStoic sometimes predicts pathways where the rules invoked to fill in intermediate steps could map to multiple possible reactions. The degree of specificity of the reaction rules can be controlled by preferentially using moieties of size 3 or 2 and only size 1 if no solutions were recovered. Note that novoStoic does not allow for mixing of moieties of different sizes during the pathway design phase. As anticipated, larger moiety sizes generally yield novel steps “closer” to a known reaction and thus more likely to involve an existing (promiscuous) enzyme with some level of this activity. However, larger moiety sizes (distance of 2 or 3) severely restrict the number of possibilities for novel steps. Generally, we start the pathway design using moieties of distance 3 and then reduce to 2 or even 1 depending on the efficacy of the search so far. In addition, the requirement of elementally balanced reaction rules with proper cofactor utilization and stereo-chemical changes necessitate a high-quality biochemical database as an input for rePrime/novoStoic. Incomplete or incorrect reaction annotation (e.g., molecular structure, stereochemistry, stoichiometry, cofactor, and reaction mechanism) could significantly affect the quality of the rules identified and the reliability of a pathway. A number of automated algorithms^60^ and procedures^61^ have been developed to reduce annotation inconsistencies and unify discrepancies across different databases. However, expert curation is often necessary to include updated discoveries (e.g., fixing cofactor utilization of a stoichiometrically balanced reaction) as well as to evaluate and resolve contradicting information^62^. Furthermore, we generally treat all rules as reversible. Therefore, additional scrutiny may be needed to ensure that the reaction rule ultimately maps to a reaction that is thermodynamically feasible.

Moving forward, the rePrime/novoStoic framework can be augmented by ranking designed pathways based on additional criteria on enzyme performance, toxicity of intermediate metabolites coupled with genetic intervention tools to explore high-yield reaction modulations or deletions^63–65^. Machine learning-based algorithms such as support vector machine and Gaussian processes have already been applied to select protein sequences based on their predicted probability of promiscuity and enzyme–substrate affinity^59, 66^. Databases such as Tox21^67^ can be used to predict the lethality of pathway intermediates and thus filter accordingly pathway designs. In addition, the pathways can be ranked based on their orthogonality score^68^ when exploring genetic intervention strategies after the pathway/reaction addition in the production host are made. The addition of new pathways designed by rePrime/novoStoic or other methods will increase the probability of orthogonality^68^ by expanding upon the range of alternative pathways and possibly destroy growth-coupled mode of production.

## Methods

### Data and parameters required by rePrime and novoStoic

Reactions and metabolites from KEGG, BRENDA, MetaCyc, Rhea, HMDB, ECMDB, ChEBI, and ChEMBL and over 112 metabolic models were aggregated and standardized to create a database with elementally balanced reactions using the MetRxn curation workflow^19^. Note that the application of unbalanced reactions should always be avoided as they would generate elementally unbalanced rules thus leading to incorrect predictions. rePrime/novoStoic requires as input: (i) the standardized data set, which contains 44,784 unique elementally balanced reactions and 32,478 metabolites encoded within sets *J* and *I*, respectively, (ii) molecular graph of each metabolite (include cofactors), wherein each atom was represented by a node *n* that is indexed uniquely in the set ${\Bbb N}_i$, (iii) formation energies of exchange metabolites that were calculated using component contribution method^69^ or eQuilibrator^70^ (at standard cellular conditions, pH 7.0, and ionic strength of 0.1 M) and stored as ${\mathrm{\Delta }}_{\mathrm{f}}G_i^{\prime \circ }$, and (iv) the reactions in pathway/subsystem (set ${\Bbb P}$) and organism/genus/taxa (set B) annotations downloaded from KEGG and stored as sets *J*_P_ and *J*_B_, respectively.

### Developing a database of reaction rules using rePrime

The rePrime procedure uses the information encoded in the molecular graphs to generate molecular signatures and subsequently reaction rules. Principles from existing reaction rule operators BEM^23^, RDM^24^, and SMIRKS^25^ schemes were integrated into rePrime. BEM tracks bond changes as a summation operator, whereas RDM codifies the topological changes in the neighborhood of the reaction center, as changes between chemical fingerprints. The SMIRKS protocol leverages prime factorization to codify molecular structures as canonical strings. In rePrime, all nodes in each molecular graph were first assigned canonical labels and ranked (Fig. 2). Each molecular graph was then represented by a vector called molecular signature that captures the count of unique nodes (i.e., moiety centered at node *n*) identified by their canonical labels. Molecular signatures provide a convenient data structure for elementary graph operations. Graph edit operations such as addition and/or subtraction to transform the molecular signatures of reactants into products capture the bond changes between reactants and products. By linking bond changes to the reactant-product molecular signature of cataloged reactions, we can identify generalized reaction rules primitives shared by multiple reactions.

The rePrime procedure to generate these reaction rules can be divided into three main steps (see Fig. 2, Supplementary Table 4, and Supplementary Methods for algorithmic details).

Step 1: Each node on a molecule is first assigned a canonical label. Note that the canonical label can be extended with stereo-descriptors as described in the CLCA^34^ algorithm to account for stereo-chemical changes when the goal of a pathway design involves such changes. A prime number is then assigned iteratively to each node for every metabolite (including cofactors), wherein each prime number maps uniquely to a specific moiety.

Step 2: The molecular signature $C_{mi}^\lambda$ (i.e., a vector containing the number of moieties) for each metabolite (including cofactors) is extracted.

Step 3: The reaction rule termed $T_{mj}^\lambda$ for each reaction in the database is derived based on the stoichiometry of the reaction and the molecular signatures of the participating metabolites. A unique set of reaction rules $T_{mr}^\lambda$ is then retained to generate the reaction rule database (Fig. 1). Every rule is currently assumed to be reversible. However, additional constraints can be added to restrict the reversibility of reaction based on experimental evidence or Gibbs free energy of reaction (e.g., component contribution method^69^).

Steps 1–3 were repeated for different moiety sizes (*λ *= {1, 2, 3}), which represent the distance between atoms (nodes) in a molecular graph (Fig. 2a). After analyzing 44,784 (non-transport) MetRxn reactions using rePrime, a total of 826, 1929, and 6043 unique reaction rules at moiety size (*λ*) of 1, 2, and 3, respectively, were extracted (see Supplementary Methods for details). The database for reaction rules was then used within the novoStoic procedure to predict the route to product molecules from reactant molecules. rePrime is consistent with the algorithmic requirements (i.e., elementally balanced reaction rules) of the novoStoic pathway prediction procedure.

### Pathway design by using the novoStoic optimization framework

The novoStoic procedure implements an MILP optimization framework to identify a metabolite- and moiety-balanced biochemical pathway that converts source to target molecule(s) (Fig. 1). In particular, the novoStoic algorithm searches within a merged database of both MetRxn reactions and rePrime reaction rules. Preference is given to the use of reactions while reaction rules are minimally invoked when there is not a cataloged reaction available to complete the pathway. The objective function generally involves the maximization of the profit margin (i.e., the cost difference between substrate and product) as a way of prioritizing toward biosynthetic routes from inexpensive substrates to high-value products. The objective function can be modified to the minimization of the number of reaction rules or known reaction step(s) that form the pathway. By design, the pathway is component and moiety-balanced with an overall standard free energy of change that is negative. The combined use of reaction rules expands the reaction search space with putative enzyme-catalyzed reactions that may be realized through protein engineering for the desired substrate specificity. Therefore, novoStoic could explore de novo biosynthetic or biodegradation routes, which are yet to be catalogued in the enzyme databases. The novoStoic algorithm is versatile enough to allow for any combination of the design rules such as customization of network size, selection of host organism to minimize heterologous reactions, and enforcing the selection of reactions/rules from common categories (see Supplementary Methods for details).

### Code availability

The code supporting the findings of this study is available at https://github.com/maranasgroup as Python and GAMS scripts. The Git repositories for rePrime and novoStoic will be made public post publication.

### Data availability

All data generated or analyzed during this study are included in this published article and on MetRxn database (http://www.maranasgroup.com/metrxn).

## Electronic supplementary material

Supplementary Information
