# Supplementary material for: Pathway design using de novo steps through uncharted biochemical spaces
Source: Nat Commun. 2018 Jan 12;9:184. doi: 10.1038/s41467-017-02362-x (PMC5766603; doi:10.1038/s41467-017-02362-x)
Supplement: Supplementary file 1 — Supplementary Information [file 41467_2017_2362_MOESM1_ESM.pdf]

Supplementary Methods

The rePrime algorithm workflow

The rePrime algorithm is used to generate molecular signatures and derive reaction rules from a database of known reactions (i.e. MetRxn database[1]). A molecular signature is a vector that concatenates into a single value the number of attributes for each moiety in a molecule, described as a collection of prime numbers (see Fig. 2). A reaction rule is thus defined as the vector that captures the changes in all moieties of participating metabolites upon reaction. A reaction rule uniquely captures the eliminated and newly formed moieties around the reaction center. rePrime involves three major steps that are iteratively applied for an increasing moiety size (λ) (i.e., distance in graph) centered at node n. At the beginning ($\lambda = 1$), the moiety is simply the atom at node n, whereas after one iteration ($\lambda = 2$) the moiety is composed of all the atoms bonded to the atom at location n. At $\lambda = 3$, the moiety encompasses all atoms connected by at most two bonds with node n. Typically, we terminate for $\lambda = 3$ as this yields reaction rules that most economically capture the diversity of available reactions in the database (see Fig. 2a). A detailed description of using rePrime to generate molecular signatures and reaction rules is provided in the following sections. A toy example demonstrates the rePrime algorithm using a set of two decarboxylase reactions i.e., 2-hydroxyisophthalate decarboxylase (2HIPD) and salicylate decarboxylase (SLD) (see Supplementary Fig. 1).

Step 1: Identification of moieties for all metabolites in the MetRxn database

rePrime is applied to the MetRxn database that is comprised of a set $I = \{i|1, \dots, M\}$ of metabolites and a set $J = \{j|1, \dots, N\}$ of reactions. For each metabolite, a node set $\mathbb{N}_i = \{n|1, \dots, Q\}$ denotes the list of atoms (see Supplementary Fig. 1). To capture the context-specific nature of each atom n in a metabolite i, atom-feature (K_{ni}) is defined as a string that encodes information about the atom and bonding environment. For example, the atom feature "3-4-06-0" for the carbon atom at location $n = 3$ of 2-hydroxyisophthalate (see Fig. 2b) encodes the presence of 3 non-hydrogen connections, 4 non-hydrogen bonds, the atomic number of carbon (i.e., 6), and 0 hydrogen bond, respectively. Since no reactions involved stereochemistry changes in our case studies, we chose here to use a simplified atom feature string for the simplicity of illustration. Note that the atom-feature is an option in rePrime that can be extended to include more descriptors depending on the goal of the users. For example, to discover pathways that account for stereochemical changes, the atom feature string can be extended to include the stereo descriptor as described in the CLCA[2] algorithm. At moiety size $\lambda = 1$, the rePrime procedure initiates by identifying the unique moieties in all metabolites, and assigning a prime number label uniquely representing a particular moiety in an ascending order of the lexical order of the string K_{ni}.

In detail, the first ($\lambda = 1$) assignment of prime numbers is performed as followed:

$$P_{ni}^{\lambda} \leftarrow \boldsymbol{h}: K_{ni} \qquad \forall n \in \mathbb{N}_i, \forall i \in I, \lambda = 1 \qquad (1.1)$$

where P_{ni}^{λ} is the prime number assigned for atom n in metabolite i and moiety size λ, K_{ni} is the atom-feature for atom n of metabolite i, and $\boldsymbol{h}$ is an injective function that maps K_{ni} to a unique prime number based on the lexical ordering in an ascending order of the atom-features K_{ni}. Consequently, function $\boldsymbol{h}$ assigns a unique prime number in a rank-ordered manner. Thus, nodes 3, 4, 8, 9 and 12 in molecule 2-hydroxyisophthalate sharing the same atom-feature "3-4-06-0"

are assigned the same 5th prime number '11' in accordance with the lexical ordering in the column of atom-features (see Fig. 2b and Supplementary Table 1). Supplementary Table 1 shows the atom features and their corresponding moieties at $\lambda = 1$ (i.e. P_{ni}^{1}) for all the nodes of metabolites 2-hydroxyisophthalate (2hipa), salicylate (sal), phenol (phnl) and carbon dioxide (co_2).

rePrime next expands the range to $\lambda = 2$, and recalculates the unique label of each atom and the corresponding prime number assignments (i.e. P_{ni}^{2}). As described in CLCA algorithm[2], the procedure first applies prime-factorization to generate a unique canonical label for each atom and then maps it with a unique prime number. Note that rePrime does not involve atom mapping but applies the atom-feature string described in the atom mapping algorithm CLCA[2] to extract the number of moieties and combined them into a molecular signature representing each metabolite. The unique canonical label is the product of prime numbers assigned to the adjacent atoms in the previous iteration and is generated as follows:

$$Z_{ni}^{\lambda} \leftarrow (P_{ni}^{\lambda})^2 \prod_{n^* \in A_{ni}} P_{n^*i}^{\lambda} \qquad \forall n \in \mathbb{N}_i, \forall i \in I, \lambda = 1 \qquad (1.2)$$

where Z_{ni}^{λ} stores the prime-product for each node n of metabolite i at moiety size λ, and A_{ni} is the set of adjacent nodes of atom n in metabolite i. The prime-product is composed of the primes assigned to atom n and all the atoms connected with n (i.e., n^*). Therefore, it is informed by the properties of all the atoms connected to the atom at location n. Figure 2c depicts the prime-product calculated for each node in the metabolite 2hipa. The adjacent nodes $n^* \in \{1, 2, 4\}$ of node $n = 3$ are assigned the prime numbers 3, 2 and 11, and hence $Z_{3,2hipa}^{2}$ calculated in step (1.2) is equal to '7986'. Note that the same prime-product '7986' is obtained for atom $n = 9$ of hipa and $n = 16$ of sal (see Supplementary Table 1) indicating the presence of a carboxyl group (-COOH) at those locations on both the molecules 2hipa and sal. The proposed labeling scheme recognizes common moieties within a moiety size of λ and assigns to them the same prime-product canonical label designation.

Next, we map a unique prime number to each canonical label Z_{ni}^{λ} by the injective function $\boldsymbol{h}$:

$$P_{ni}^{\lambda+1} \leftarrow \boldsymbol{h}: Z_{ni}^{\lambda} \qquad \forall n \in \mathbb{N}_i, \forall i \in I, \lambda = 1 \qquad (1.3)$$

Similar to equation (1.1), function $\boldsymbol{h}$ maps prime numbers based on the lexical ordering in an ascending order of canonical label Z_{ni}^{λ}. For example, $P_{3,2hipa}^{2}, P_{9,2hipa}^{2}$ and $P_{16,sal}^{2}$ are assigned the same 8th prime number '19' based on the Z_{ni}^{λ} value of '7986' (see Fig. 2c and Supplementary Table 1). In the next iteration, (i.e., moiety size $\lambda = 3$) equations (1.2) and (1.3) are applied again to calculate P_{ni}^{3}. Similarly, P_{ni}^{3} captures the moieties that encompasses all atom connected by at most two bonds with atom n. This step recognizes the presence of the larger common groups between molecules 2hipa and sal. Supplementary Table 1 shows the values for P_{ni}^{λ} and Z_{ni}^{λ} for each $\lambda \in \{1,2,3\}$ and for every atom of each metabolite involves in reactions 2HIPD and SLD. All prime numbers (P_{ni}^{λ}) are ordered into the set $\mathbb{M}^{\lambda} = \{m|2,3,5,7,11 \ldots\}$ to index each moiety m (see Supplementary Table 2).

Step 2: Determination of the molecular signature of each metabolite.

The molecular signatures are assembled by counting the number of each moiety m in each metabolite i (see Fig. 2b and Fig. 2c):

$$C_{mi}^{\lambda} \leftarrow \sum_{n \in N_i} \delta_{P_{ni}^{\lambda}, m} \qquad \forall i \in I, \forall m \in \mathbb{M}^{\lambda}, \forall \lambda \in \{1, 2, 3\} \qquad (1.4)$$

where the Kronecker delta enables making the appropriate matches

$$\delta_{P_{ni}^{\lambda}, m} = \begin{cases} 1, \text{if } P_{ni}^{\lambda} = m \\ 0, \text{otherwise} \end{cases}$$

and C_{mi}^{λ} is the molecular signature that encodes the number of moieties m of a moiety size λ in each metabolite i. For the moieties m indexed over the domain of prime numbers $\mathbb{M}^{\lambda}$, a value of 1 is returned for each node $n \in N_i$ when $P_{ni}^{\lambda} = m$. Supplementary Table 2 shows the molecular signature of each metabolite in reactions 2HIPD and SLD. Moieties at different moiety size λ are stored in our database separately.

Step 3: Inference of the associated reaction rule for each reaction in MetRxn.
Upon a biochemical transformation, moieties present in a reactant(s) that are part of the reaction center undergo changes while the remaining moieties are left unchanged. To derive the reaction rules, we calculate the changes in the number of moieties between the reactants and the products for all reactions:

$$T_{mj}^{\lambda} \leftarrow \sum_{i \in I} S_{ij}\, C_{mi}^{\lambda} \qquad \forall j \in J, \forall m \in \mathbb{M}^{\lambda}, \forall \lambda \in \{1, 2, 3\} \qquad (1.5)$$

where each m represents a moiety and T_{mj}^{λ} is the reaction rule that encodes the change in the number of moieties m upon reaction j, and S_{ij} is the stoichiometric coefficient of metabolite i in reaction j. In equation (1.5), moieties that participate in the reaction center do not cancel out (see Fig. 2d).

Upon generating all the reaction rules, repetitive reaction rules are removed to form a unique set for the final database as follows:

$$T_{mr}^{\lambda} \leftarrow \boldsymbol{f}: T_{mj}^{\lambda} \qquad \forall j \in J, \forall m \in \mathbb{M}^{\lambda}, \forall \lambda \in \{1,2,3\} \qquad (1.6)$$

where function $\boldsymbol{f}$ removes repeated entries in T_{mj}^{λ} and assigns a new index r to each unique reaction rule. A new set $R^{\lambda} = \{r | 1, 2, \ldots, R\}$ of reaction rules is defined to index every unique reaction rule. For example, the reaction rules for reactions 2HIPD and SLD are identical and thus only one of them should remain in the database (i.e., $T_{m,2HIPD}^{1}$ and $T_{m,SLD}^{1}$ are now stored as $T_{m,1}^{1}$). Supplementary Table 3 shows the reaction rules for reaction 2HIPD and SLD at different moiety size λ. The algorithmic description of rePrime (equation 1.1 to 1.6) is summarized in Supplementary Table 4. Every rule is assumed to be reversible in the current study by hypothesizing that *de novo* enzymes can catalyze the opposite direction by creating the appropriate metabolite concentration imbalance. However, as a target of future work, a pre-processing group contribution approach that was employed in the optStoic[3] framework can be introduced into rePrime workflow to determine *a priori* the reaction reversibility.

Note that rePrime generated reaction rules also include the information of the moiety changes between cofactor pairs. For example, rePrime can distinguish reaction rules with or without ATP

(see Supplementary Table 5 and 6) because ATP and ADP are also considered as reactants and products in step 1 and step 2, and the moiety change between ATP and ADP is included in the reaction rule generated in step 3. However, in most cases, different cofactor pairs which exchange the same chemical groups (e.g., both $NADH/NAD^+$ and $NADPH/NADP^+$ transfer electrons) undergo the same change in moiety balance. As a result, the same rule can be associated with different cofactor pairs. Therefore, unless we impose a constraint on specific cofactor type that can be utilized in the pathway (e.g. the cofactors utilization constraints imposed in the BDO synthesis example), rePrime/novoStoic will not be able to differentiate between them. In such a case, we usually report the cofactor pair as NAD(P)H.

Upon the termination of the rePrime procedure, 50 unique moieties and 826 reaction rules at moiety size one, 298 unique moieties and 1,929 reactions rules at moiety size two, and 1,110 unique moieties and 6,043 reactions rules at moiety size three are calculated and stored in the database. C_{mi}^{λ} and T_{mr}^{λ} are parameters used as input data for the novoStoic optimization formulation for novel pathway design discussed in the next section.

The novoStoic algorithm: Definition of sets, parameters, variables and optimization formulation

novoStoic uses an MILP representation to pose the task of identifying a biochemical pathway that converts a source metabolite to a target as an optimization problem. The objective function involves the maximization of a profit function (e.g., the cost difference between substrate and product) to prioritize biosynthesis routes from inexpensive substrates to a high-value product. Additional requirements on the maximum number of reaction rules or reaction steps can be imposed as constraints. The identified pathways are by design component and moiety balanced. novoStoic can incorporate any combination of the following five design rules:

i) combine both known and hypothetical reactions within a component balanced and moiety balanced reaction network,
ii) customize the network size (i.e., the number of known and hypothetical reactions),
iii) select a host organism such that the least number of heterologous reactions are identified (the organism is suggested if one is not provided *a priori*),
iv) ensure that the reactions and rules belong to common categories (i.e., the same pathway or subsystem annotations in databases), and
v) ensure negative overall standard Gibbs free energy change.

The novoStoic draws from the definition of the following sets, parameters and variables.

Sets

$\mathbb{M} = \{m\|2,3,5,7,11 \dots\}$	set of moieties (prime numbers)
$R^1 = \{r\|1,2,\dots,R_1\}$	set of reaction rules calculated at moiety size $\lambda = 1$
$R^2 = \{r\|1,2,\dots,R_2\}$	set of reaction rules calculated at moiety size $\lambda = 2$
$R^3 = \{r\|1,2,\dots,R_3\}$	set of reaction rules calculated at moiety size $\lambda = 3$
$I = \{i\|1,\dots,M\}$	set of all metabolites in MetRxn
$J = \{j\|1,\dots,N\}$	set of reactions in MetRxn excluding exchange reactions
$B = \{b\|B_1,B_2,\dots,B_b\}$	set of organisms in MetRxn
$\mathbb{P} = \{p\|P_1,P_2,\dots,P_p\}$	set of pathway/subsystem annotations in KEGG, MetaCyc, and BRENDA
$J_p \subset J$	subset of reactions in pathway/subsystem p
$J_b \subset J$	subset of reactions in organism b
$I_{target} \subset I$	set of target metabolites
$I_{source} \subset I$	set of predetermined precursors that can be used to produce target metabolites
$I_{co\_metab} \subset I$	set of co-substrates/co-products allowed in the pathway design
$I_{ex} = I_{target} \cup I_{source} \cup I_{co\_metab}$	set of all exchange metabolites

Parameters

λ	moiety size, and $\lambda \in \{1,2,3\}$
S_{ij}	stoichiometric coefficient of metabolite i in reaction j
C_{mi}^{λ}	molecular signature of metabolite i encoding the number of moieties m at moiety size λ
T_{mr}^{λ}	reaction rule r that captures the change in the number of moieties m at moiety size λ
$Cost_i$	cost per mole of metabolite i

ΔG_i^f free energy of formation of metabolite i at typical cellular conditions (i.e. pH 7.0 and ionic strength of 0.1M), identified using eQuilibrator[4]

W W^{htg}: the maximum number (cutoff) of heterologous reactions in the designed network;
W^{hyp}: the maximum number of hypothetical reactions in the designed network;
W^{rxn}: the maximum number of known reactions in the designed network;
W^{path}: the maximum number of rules not associated with pathway p that can be active in the designed network

ΔG^{max} the maximum allowed value of free energy change under typical cellular conditions for the network (at pH 7.0 and ionic strength of 0.1M)

M big M (large positive constant)

S^{max} upper bound for the summation of stoichiometric coefficient of source metabolites

Continuous variables

v_j flux of reaction j

Binary variables

$$y_j^{rxn} = \begin{cases} 1, \text{if reaction } j \text{ participates in the designed pathway} \\ 0, \text{otherwise} \end{cases}$$

$$y_r^{rule} = \begin{cases} 1, \text{if reaction rule } r \text{ participates in the designed pathway} \\ 0, \text{otherwise} \end{cases}$$

$$y_b^{org} = \begin{cases} 1, \text{if organism } b \text{ is selected as the host} \\ 0, \text{otherwise} \end{cases}$$

$$y_p^{path} = \begin{cases} 1, \text{if pathway or substytem } p \text{ is selected as template for the designed pathway} \\ 0, \text{otherwise} \end{cases}$$

Integer variables

v_i^{EX} flux of the exchange reaction of metabolite i. A negative and positive value indicates the active uptake and export of metabolite i, respectively

v_r flux of a hypothetical reaction guided by reaction rule r, and a non-zero value indicates that this hypothetical reaction participates in the designed pathway

v_i^{imb} surplus or deficit of metabolite i in the known metabolic network. A positive value indicates that metabolite i is exported from the known metabolic network to the hypothetical (i.e. reaction rules) network, whereas a negative value indicates that metabolite i is imported from the hypothetical network into the known metabolic network (see Fig. 3a)

novoStoic (MILP formulation for biosynthesis network design)

The novoStoic formulation maximizes the profit margin of the overall conversion while imposing the five design rules described earlier. Note that moiety size λ is specified for each novoStoic simulation such that reaction rules and molecular signatures derived at different moiety sizes are not mixed.

$$Maximize \sum_{i \in I_{ex}} Cost_i \, v_i^{EX}$$

subject to:

$$v_i^{EX} \geq 1 \qquad \forall\, i \in\, I_{target} \qquad (1.1)$$

$$-S^{max} \;\leq \sum_{i \in I_{source}} v_i^{EX} \leq\, -1 \qquad (1.2)$$

$$\sum_{j \in J} S_{ij} v_j = v_i^{imb} \qquad \forall\, i \in\, I \qquad (2)$$

$$\sum_{r \in R^{\lambda}} T_{mr}^{\lambda} v_r + \sum_{i \in I} C_{mi}^{\lambda} v_i^{imb} = \sum_{i \in I_{ex}} C_{mi}^{\lambda} v_i^{EX} \qquad \forall\, m\, \in \mathbb{M} \qquad (3)$$

$$y_j^{rxn} LB_j \leq v_j \leq y_j^{rxn} UB_j \qquad \forall\, j \in\, J \qquad (4)$$

$$\sum_{j \in J} y_j^{rxn} \leq W^{rxn} \qquad (5)$$

$$y_r^{rule} LB_r \leq v_r \leq y_r^{rule} UB_r \qquad \forall\, r \in\, R^{\lambda} \qquad (6)$$

$$\sum_{r \in R^{\lambda}} y_r^{rule} \leq W^{hyp} \qquad (7)$$

$$\sum_{i \in I_{ex}} \Delta G_i^f\, v_i^{EX} \leq\, \Delta G^{min} \qquad (8)$$

$$v_j \in \mathbb{R} \qquad \forall\, j \in J$$

$$v_r \in \mathbb{Z}^{\mathbf{0+}} \qquad \forall\, r \in R^{\lambda}$$

$$v_i \in \mathbb{Z} \qquad \forall\, i \in I$$

$$v_i^{EX} \in \mathbb{Z}^{\mathbf{0+}} \qquad \forall\, i \in I_{target}$$

$$v_i^{EX} \in \mathbb{Z}^{\mathbf{0-}} \qquad \forall\, i \in I_{source}$$

$$v_i^{EX} \in \mathbb{Z} \qquad \forall\, i \in I_{co\_metab}$$

$$\lambda \in \{1,2,3\}$$

novoStoic maximizes the difference in prices between the substrates and targets scaled by the corresponding stoichiometric coefficients in the overall conversion. Constraint (1.1) defines the target metabolite(s), which are part of the input. Constraint (1.2) ensures that novoStoic selects only substrate molecule(s) from a list of predetermined precursors to design pathways to the targets (see Supplementary Fig. 2 for toy example). Assume that the overall conversion is

$$aA + bB + cC \rightarrow dD + eE$$

where a, b, c, d, and e are the stoichiometric coefficient of metabolites A, B, C, D, and E, respectively. These coefficients are defined as integers. Note that the exact stoichiometric coefficients do not need to be determined *a priori* as an input for novoStoic. It is only required to

specify that d is a positive integer value if D is the target metabolite, and that the summation of a and b is a negative integer if A and B are within a list of source metabolite candidates (see constraint 1.2). In the latter case, novoStoic will select A or/and B to be the source metabolite(s) (e.g., $a = -1$ and $b = 0$ if A is identified as the only source metabolite). The value of c and e can be either positive or negative depending on whether they are co-substrates or co-products identified by novoStoic. novoStoic optimizes an overall stoichiometry for the most cost-effective design. The exchange flux of the source and target metabolites of the designed pathway must conform to the overall stoichiometry as followed:

$$v_A^{EX} = -a, v_B^{EX} = -b, v_C^{EX} = -c, v_D^{EX} = d, v_E^{EX} = e$$

Constraints (2) and (3) are central for combining both known reactions and reaction rules within a single component-balanced and moiety-balanced framework. Constraint (2) enforces a stoichiometric balance in the known reaction network for each metabolite i. Any deficit or surplus of a metabolite i generated from the known reaction network is characterized by the variable v_i^{imb}. Therefore, v_i^{imb} links the known reaction network and the hypothetical reaction network (see Fig. 3). A positive value for v_i^{imb} indicates that the designed pathway carries flux from a known reaction to a hypothetical reaction at metabolite i. Likewise, a negative value for v_i^{imb} indicates that a hypothetical reaction passes flux back to a known reaction at metabolite i. All the reactions v_j in constraint (2) are internal, and thus the metabolites in the known reaction network can only be exchanged through v_i^{imb}. Constraint (3) enforces a moiety balance in the designed network for each moiety m on all v_r, v_i^{imb}, and v_i^{EX} (see Fig. 3). novoStoic searches from both known reactions and reaction rules to balance the number of moieties. The right hand side of constraint (3) ($\sum_{i \in I_{ex}} C_{mi}^{\lambda} v_i^{EX}$) represents the overall moiety change of the designed pathway for each moiety m. The first term on the left hand side ($\sum_{r \in R^{\lambda}} T_{mr}^{\lambda} v_r$) indicates the total moiety changes in all the hypothetical reactions for each moiety m, whereas the second term ($\sum_{i \in I} C_{mi}^{\lambda} v_i^{imb}$) depicts the total moiety changes in all the known reactions for each moiety m. Thus, the overall moiety change equals the summation of moiety changes in both the hypothetical and known reactions. When $\sum_{i \in I} C_{mi}^{\lambda} v_i^{imb} \neq \sum_{i \in I_{ex}} C_{mi}^{\lambda} x_i^{EX}$, the term $\sum_{r \in R^{\lambda}} T_{mr}^{\lambda} v_r$ must be active thus implying that certain reaction rules are selected into the designed pathway. As shown in the toy example (see Supplementary Fig. 2), using the component/moiety balance constraints, the solution (i) selects two known reactions (2HIPD and SLD) to produce phnl and no hypothetical reactions are involved ($v_{R1} = 0$), whereas solution (iv) selects only reaction rules and no known reactions are involved ($v_{R1} = 2$). Constraints (4) and (5) control the number of known reactions allowed into the designed network, whereas constraints (6) and (7) control the number of reaction rules allowed into the designed network. In the toy example, we define that the maximum number of known reactions $W^{rxn} = 2$ and the maximum number of hypothetical reactions $W^{hyp} = 2$ to control the network size. Constraint (8) forces the overall conversion to have a negative standard free energy change. The parameters needed for this constraint (ΔG_i^f) were calculated using eQuilibrator[5].

More constraints are active when we design more complex pathways to reduce the search space. With these additional constraints, novoStoic identifies the suitable chassis organism for engineering and simultaneously selects reactions predominantly from a particular reaction category (i.e., pathway/subsystem). Constraints (8) and these additional constraints are not active for the toy example.

$$\sum_{j \in J \backslash J_b} y_j^{rxn} \leq y_b^{org} W^{htg} + \left(1 - y_b^{org}\right) M \qquad \forall\, b \in B \qquad (9)$$

$$\sum_{b \in B} y_b^{org} = 1 \qquad (10)$$

$$\sum_{j \in J \backslash J_p} y_j^{rule} \leq y_p^{path} W^{path} + \left(1 - y_p^{path}\right) M \qquad \forall\, p \in \mathbb{P} \qquad (11)$$

$$\sum_{p \in P} y_p^{path} = 1 \qquad (12)$$

In detail, constraints (9) and (10) cause the selection of the maximum number of reactions from organism b and minimize the number of heterologous reactions (i.e. reactions not from organism b are controlled by W^{htg}). Constraints (11) and (12) ensure the reactions and rules belong to common categories. The categories defined by pathway and subsystem annotations in databases such as KEGG[6], MetaCyc[7] and BRENDA[8] are manually curated by experts and built on the observation that certain sequence of chemical transformations is conserved across various species and taxa. Genetic loci (gene clusters) and genetic controls related to expression and regulation have also been factored into the pathway and subsystem annotators[9].

In addition to prospecting for biosynthetic pathways, novoStoic can be applied to identify degradation pathways for a molecule. In this case, we set the objective function to identify the minimal set of reactions and reaction rules needed to degrade a source molecule to a target molecule as followed:

$$Minimize \sum_{j \in J} y_j^{rxn} + \sum_{r \in R} y_r^{rule}$$

To design a biodegradation pathway, both the source metabolite(s) and target metabolite(s) are predetermined. Thus, constraint (1.2) is changed accordingly:

$$v_i^{EX} \leq -1 \qquad \forall\, i \in\ I_{source} \qquad (1.2)$$

The other constraints (1.1, 2 to 12) are also applied in the formulation for the degradation studies. Similar to the biosynthesis formulation, non-zero values of v_j and v_r indicates the participation of reaction j and rule r in the designed pathway. Other than the exchange reactions for the predetermined source metabolite(s) (v_{source}^{EX}) and target metabolite(s) (v_{target}^{EX}), a positive value of v_i^{EX} indicates that metabolite i is the co-product to the target, and a negative value of v_i^{EX} indicates metabolite i is the co-substrate. The oxidative degradation of benzo[a]pyrene to catechol in the result section provides a detailed implementation of using novoStoic to develop degradation pathways.

rePrime/novoStoic avoids the pre-generation of extremely large metabolic networks and requires the use of a MILP solver, which is currently made available freely (by Gurobi, Inc and IBM) for academic use. The largest pathway size that we have attempted so far using rePrime/novoStoic is 21 steps as described in the case study of benzo[a]pyrene degradation. However, the

computational time is not determined solely by the pathway length. Multiple factors could also contribute to the complexity of a pathway design, such as (i) moiety size, (ii) whether the desired pathway involves hub molecules (e.g., pyruvate or succinate) that are associated with many reaction rules, and (iii) the number of isomorphic regions in the starting substrates (a rule can act on multiple regions and produce different compounds).

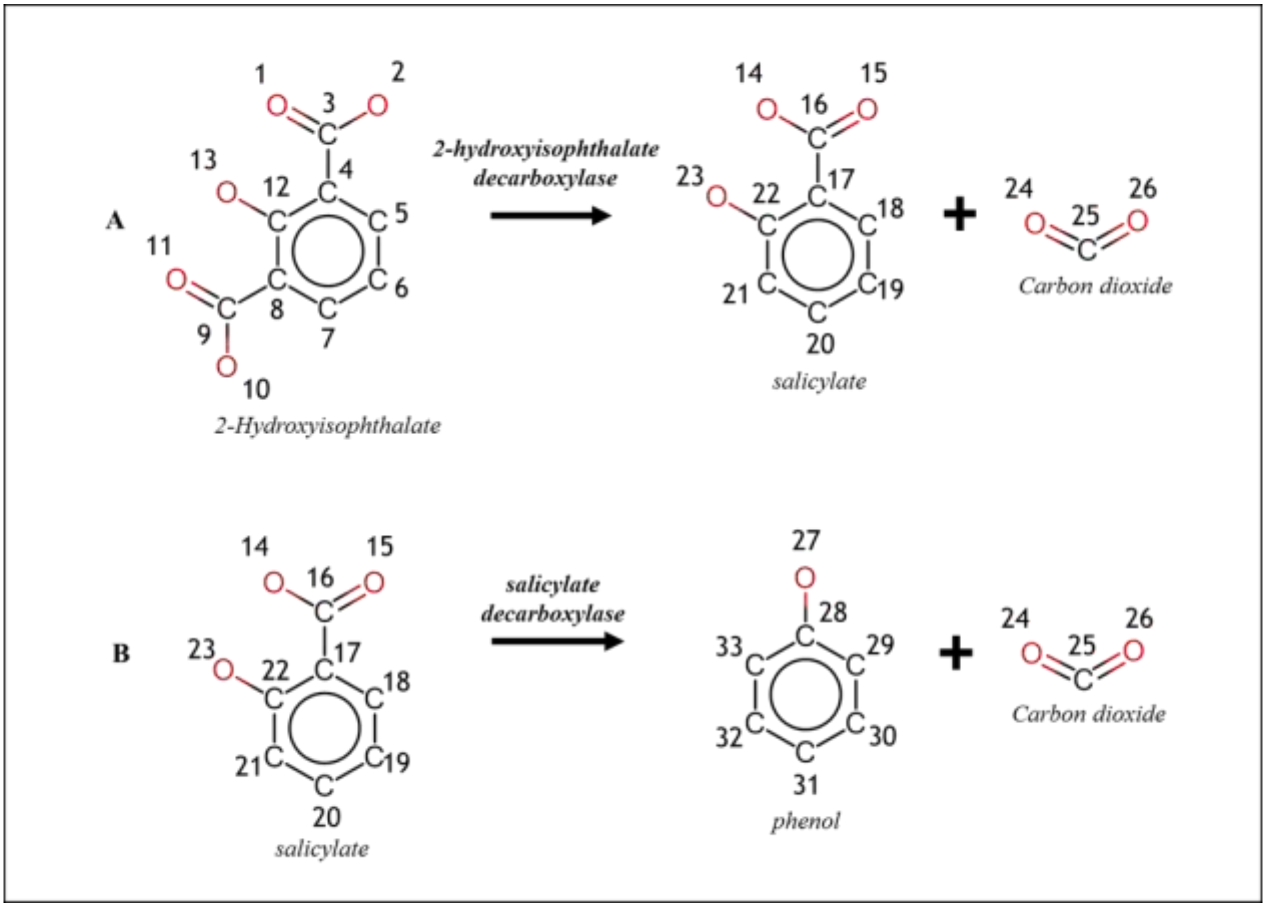

Supplementary Figure 1: **Graph representation of reaction *2-hydroxyisopthalate decarboxylase* (2HIPD) and *salicylate decarboxylase* (SLD).** Each graph has a node set $\{n|1, \ldots, Q\}$ that denotes the list of atoms composing the metabolites. Each unique metabolite within the MetRxn database has the same node set regardless of the reactions in which it participates. For example, the metabolite salicylate has the same node set {14, 15, …, 23} in both reactions 2HIPD and SLD.

$$\textit{Maximize}\quad price_{2hipa}\cdot v_{2hipa}^{EX} + price_{phnl}\cdot v_{phnl}^{EX} + price_{co2}\cdot v_{co2}^{EX}$$

s.t.

$$\left.\begin{array}{l} v_{phnl}^{EX} \geq 1 \\ v_{2hipa}^{EX} \leq -1 \end{array}\right\} \quad (1)$$

$$\left.\begin{array}{l} -v_{2HIPD} = v_{2hipa}^{imb} \\ v_{2HIPD} - v_{SLD} = v_{sal}^{imb} \\ v_{2HIPD} + v_{SLD} = v_{co2}^{imb} \\ v_{SLD} = v_{phnl}^{imb} \end{array}\right\} \quad (2)$$

$$v_{R1}\begin{bmatrix}-1\\1\\1\\1\\-2\end{bmatrix} + v_{2hipa}^{imb}\begin{bmatrix}3\\2\\3\\0\\5\end{bmatrix} + v_{sal}^{imb}\begin{bmatrix}2\\1\\4\\0\\3\end{bmatrix} + v_{co2}^{imb}\begin{bmatrix}0\\2\\0\\1\\0\end{bmatrix} + v_{phnl}^{imb}\begin{bmatrix}1\\0\\5\\0\\1\end{bmatrix} = v_{2hipa}^{EX}\begin{bmatrix}3\\2\\3\\0\\5\end{bmatrix} + v_{co2}^{EX}\begin{bmatrix}0\\2\\0\\1\\0\end{bmatrix} + v_{phnl}^{EX}\begin{bmatrix}1\\0\\5\\0\\1\end{bmatrix} \quad (3)$$

$$\left.\begin{array}{l} y_{2HIPD}^{rxn}\cdot LB_{2HIPD} \leq v_{2HIPD} \leq y_{2HIPD}^{rxn}\cdot UB_{2HIPD} \\ y_{SLD}^{rxn}\cdot LB_{SLD} \leq v_{SLD} \leq y_{SLD}^{rxn}\cdot UB_{SLD} \end{array}\right\} \quad (4)$$

$$y_{2HIPD}^{rxn} + y_{SLD}^{rxn} \leq 2 \quad (5)$$

$$y_{R1}^{rule}\cdot LB_{R1} \leq v_{R1} \leq y_{R1}^{rule}\cdot UB_{R1} \quad (6)$$

$$y_{R1}^{rule} \leq 2 \quad (7)$$

$$LB_{SLD} = LB_{2HIPD} = LB_{R1} = -1$$
$$UB_{SLD} = UB_{2HIPD} = UB_{R1} = 1$$

solutions	y_{R1}^{rule}	y_{2HIPD}^{rxn}	y_{SLD}^{rxn}	v_{R1}	v_{2hipa}^{imb}	v_{sal}^{imb}	v_{co2}^{imb}	v_{phnl}^{imb}	v_{2hipa}^{EX}	v_{co2}^{EX}	v_{phnl}^{EX}
(i)	0	1	1	0	-1	0	2	1	-1	2	1
(ii)	1	1	0	1	-1	1	1	0	-1	2	1
(iii)	1	0	1	1	0	-1	1	1	-1	2	1
(iv)	2	0	0	2	0	0	0	0	-1	2	1

Supplementary Figure 2: Expanded constraints and solutions of novoStoic for the toy example of phenol synthesis. Constraint 1 defines the source molecule and products. Constraints 2 and 3 describe the component and moiety balances using the reactions rule generated at moiety size $\lambda = 1$. Constraint 4 to 7 control the number of known reactions (i.e., 2HIPD and SLD) and reaction rules (i.e. R1 represented by $T_{m,1}^{1}$). The MILP formulation designs four alternative pathways including only known reactions (solution i) and invoking one (solution ii and iii) or two novel steps (solution iv).

Supplementary Table 1: The values of parameters P_{ni}^{λ} and Z_{ni}^{λ} assigned in the first step of rePrime to identify moieties, which are represented by prime numbers, for all metabolites in reactions 2HIPD and SLD.

i	$n \in N_i$	Atom features	P_{ni}^{1}	Z_{ni}^{1}	P_{ni}^{2}	Z_{ni}^{2}	P_{ni}^{3}
2hipa	1	1-2-08-0	3	99	5	475	17
	2	1-1-08-1	2	44	2	76	5
	3	3-4-06-0	11	7986	19	111910	47
	4	3-4-06-0	11	73205	31	6883643	67
	5	2-3-06-1	5	1375	13	57629	41
	6	2-3-06-1	5	625	11	20449	29
	7	2-3-06-1	5	1375	13	57629	71
	8	3-4-06-0	11	73205	31	6883643	67
	9	3-4-06-0	11	7986	19	111910	47
	10	1-1-08-1	2	44	2	76	5
	11	1-2-08-0	3	99	5	475	17
	12	3-4-06-0	11	29282	29	1616402	59
	13	1-1-08-1	2	44	2	116	11
sal	14	1-1-08-1	2	44	2	76	5
	15	1-2-08-0	3	99	5	475	17
	16	3-4-06-0	11	7986	19	111910	47
	17	3-4-06-0	11	73205	31	5459441	61
	18	2-3-06-1	5	1375	13	57629	41
	19	2-3-06-1	5	625	11	17303	23
	20	2-3-06-1	5	625	11	17303	23
	21	2-3-06-1	5	1375	13	42757	37
	22	3-4-06-0	11	13310	23	426374	53
	23	1-1-08-1	2	44	2	92	7
co2	24	1-2-08-0	3	63	3	63	2
	25	2-4-06-0	7	441	7	441	13
	26	1-2-08-0	3	63	3	63	2
phnl	27	1-1-08-1	2	44	2	68	3
	28	3-4-06-0	11	6050	17	97682	43
	29	2-3-06-1	5	1375	13	31603	31
	30	2-3-06-1	5	625	11	17303	23
	31	2-3-06-1	5	625	11	14641	19
	32	2-3-06-1	5	625	11	17303	23
	33	2-3-06-1	5	1375	13	31603	31

Supplementary Table 2: The values of parameter C_{mi}^{λ} assigned in the second step of rePrime to generate molecular signatures for metabolites 2hipa, sal, co2, and phnl.

	$i = 2hipa$			$i = sal$			$i = co_2$			$i = phnl$		
m	$C_{m,i}^{1}$	$C_{m,i}^{2}$	$C_{m,i}^{3}$	$C_{m,i}^{1}$	$C_{m,i}^{2}$	$C_{m,i}^{3}$	$C_{m,i}^{1}$	$C_{m,i}^{2}$	$C_{m,i}^{3}$	$C_{m,i}^{1}$	$C_{m,i}^{2}$	$C_{m,i}^{3}$
2	3	3	0	2	2	0	0	0	2	1	1	0
3	2	0	0	1	0	0	2	2	0	0	0	1
5	3	2	2	4	1	1	0	0	0	5	0	0
7	0	0	0	0	0	1	1	1	0	0	0	0
11	5	1	1	3	2	0	0	0	0	1	3	0
13	0	2	0	0	2	0	0	0	1	0	2	0
17	0	0	2	0	0	1	0	0	0	0	1	0
19	0	2	0	0	1	0	0	0	0	0	0	1
23	0	0	0	0	1	2	0	0	0	0	0	2
29	0	1	1	0	0	0	0	0	0	0	0	0
31	0	2	0	0	1	0	0	0	0	0	0	2
37	0	0	0	0	0	1	0	0	0	0	0	0
41	0	0	2	0	0	1	0	0	0	0	0	0
43	0	0	0	0	0	0	0	0	0	0	0	1
47	0	0	2	0	0	1	0	0	0	0	0	0
53	0	0	0	0	0	1	0	0	0	0	0	0
59	0	0	1	0	0	0	0	0	0	0	0	0
61	0	0	0	0	0	1	0	0	0	0	0	0
67	0	0	2	0	0	0	0	0	0	0	0	0

Supplementary Table 3: The values of parameter T_{mj}^{λ} derived in the third step of rePrime to identify reaction rules of reactions 2HIPD and SLD.

	$j = 2HIPD$			$j = SLD$		
m	T_{mj}^{1}	T_{mj}^{2}	T_{mj}^{3}	T_{mj}^{1}	T_{mj}^{2}	T_{mj}^{3}
2	-1	-1	2	-1	-1	2
3	1	2	0	1	2	1
5	1	-1	-1	1	-1	-1
7	1	1	1	1	1	-1
11	-2	1	-1	-2	1	0
13	0	0	1	0	0	1
17	0	0	-1	0	1	-1
19	0	-1	0	0	-1	1
23	0	1	2	0	-1	0
29	0	-1	-1	0	0	0
31	0	-1	0	0	-1	2
37	0	0	1	0	0	-1
41	0	0	-1	0	0	-1
43	0	0	0	0	0	1
47	0	0	-1	0	0	-1
53	0	0	1	0	0	-1
59	0	0	-1	0	0	0
61	0	0	1	0	0	-1
67	0	0	-2	0	0	0

Supplementary Table 4: Pseudo code description for rePrime algorithm output and workflow

Algorithm: rePrime

rePrime output:
molecular signature (C_{mi}^{λ}) captures moiety m in metabolite i at moiety size λ
reaction rule (T_{mr}^{λ}) captures the change of moiety m in each unique reaction rule r at moiety size λ

$P_{ni}^{\lambda} \leftarrow \boldsymbol{h}: K_{ni}$	$\forall n \in \mathbb{N}_i, \forall i \in I, \lambda = 1$
**while** $(\lambda \leq 3)$ **do**	
$\quad Z_{ni}^{\lambda} \leftarrow (P_{ni}^{\lambda})^2 \prod_{n^* \in A_{ni}} P_{n^*i}^{\lambda}$	$\forall n \in \mathbb{N}_i, \forall i \in I$
$\quad P_{ni}^{\lambda+1} \leftarrow \boldsymbol{h}: Z_{ni}^{\lambda}$	$\forall n \in \mathbb{N}_i, \forall i \in I$
$\quad \lambda = \lambda + 1$	
**End**	
$C_{mi}^{\lambda} \leftarrow \sum_{n=1}^{N_i} \delta_{P_{ni}^{\lambda}, m}$	$\forall i \in I, \forall m \in \mathbb{M}^{\lambda}, \forall \lambda \in \{1, 2, 3\}$
$T_{mj}^{\lambda} \leftarrow \sum_{i \in I} S_{ij} C_{mi}^{\lambda}$	$\forall j \in J, \forall m \in \mathbb{M}^{\lambda}, \forall \lambda \in \{1, 2, 3\}$
$T_{mr}^{\lambda} \leftarrow \boldsymbol{f}: T_{mj}^{\lambda}$	$\forall j \in J, \forall m \in \mathbb{M}^{\lambda}, \forall \lambda \in \{1, 2, 3\}$

Supplementary Table 5: Reaction rule for succinate semialdehyde dehydrogenase (the forward reaction does not use ATP as a cofactor)

	Succinate semialdehyde + H_2O +$NADP^+$ => Succinate + NADPH					
Moiety (m)	Succinate semialdehyde	H_2O	$NADP^+$	Succinate	NADPH	Reaction rule
41	0	1	0	0	0	-1
83	0	0	2	0	2	0
97	1	0	7	2	7	1
127	2	0	4	2	4	0
173	2	0	2	2	3	1
181	0	0	6	0	6	0
191	1	0	6	0	5	-2
193	0	0	3	0	3	0
229	0	0	1	0	2	1
241	1	0	13	2	13	1
251	0	0	1	0	0	-1
271	0	0	3	0	3	0

Supplementary Table 6: Reaction rule for the reverse reaction of succinate semialdehyde dehydrogenase (the reverse reaction requires ATP as a cofactor)

	Succinate + NADPH + ATP => Succinate semialdehyde + NADP$^+$ + ADP + Pi							
Moiety (m)	Succinate	NADPH	ATP	Succinate semialdehyde	NADPH	ADP	Pi	Reaction Rule
41	0	0	0	0	0	0	0	0
83	0	2	1	0	2	1	0	0
97	2	7	6	1	7	0	0	1
127	2	4	3	2	4	5	3	0
173	2	3	1	2	3	2	1	0
181	0	6	4	0	6	1	0	-1
191	0	5	2	1	5	3	0	1
193	0	3	3	0	3	2	0	0
229	0	2	1	0	2	3	0	0
241	2	13	7	1	13	1	0	-1
251	0	0	0	0	0	7	0	0
271	0	3	3	0	3	0	0	0

Supplementary References

1. Kumar, A., Suthers, P. F. & Maranas, C. D. MetRxn: a knowledgebase of metabolites and reactions spanning metabolic models and databases. *BMC Bioinformatics* **13,** (2012).
2. Sander, J. D. & Joung, J. K. CRISPR-Cas systems for editing, regulating and targeting genomes. *Nature Biotechnology* **32,** 347–55 (2014).
3. Chowdhury, A. & Maranas, C. D. Designing overall stoichiometric conversions and intervening metabolic reactions. *Sci Rep* **5,** 16009 (2015).
4. Bonde, M. T. *et al.* Direct Mutagenesis of Thousands of Genomic Targets Using Microarray-Derived Oligonucleotides. *ACS Synth Biol* **4,** 17–22 (2014).
5. Siegel, J. B. *et al.* Computational protein design enables a novel one-carbon assimilation pathway. *PNAS* **112,** 3704–3709 (2015).
6. Ogata, H. *et al.* KEGG: Kyoto Encyclopedia of Genes and Genomes. *Nucleic Acids Res.* **27,** 29–34 (1999).
7. Rahman, S. A., Advani, P., Schunk, R., Schrader, R. & Schomburg, D. Metabolic pathway analysis web service (Pathway Hunter Tool at CUBIC). *Bioinformatics* **21,** 1189–93 (2005).
8. Blum, T. & Kohlbacher, O. MetaRoute: fast search for relevant metabolic routes for interactive network navigation and visualization. *Bioinformatics* **24,** 2108–2109 (2008).
9. Rodrigo, G., Carrera, J., Prather, K. J. & Jaramillo, A. DESHARKY: Automatic design of metabolic pathways for optimal cell growth. *Bioinformatics* **24,** 2554–2556 (2008).
